# Determination of Montelukast Sodium and Bambuterol Hydrochloride in Tablets using RP HPLC

**DOI:** 10.4103/0250-474X.51961

**Published:** 2009

**Authors:** Smita Patil, Y. V. Pore, B. S. Kuchekar, Aruna Mane, V. G. Khire

**Affiliations:** Department of Pharmaceutical Chemistry, Government College of Pharmacy, Karad, 415 124, India; 1 Department of Okasa Pharma, L-2, Additional MIDC, Satara-415 004, India

**Keywords:** Montelukast sodium, bambuterol hydrochloride, HPLC, simultaneous, dosage form

## Abstract

An accurate, specific and precise assay level gradient reverse-phase high-performance liquid chromatographic method was developed for simultaneous determination of montelukast sodium and bambuterol hydrochloride in tablet dosage form. An inertsil ODS C-18, 5 μm column having 250×4.6 mm I.D. in gradient mode, with mobile phase A, containing 0.025 M sodium phosphate buffer: methanol (85:15) and mobile phase B, containing acetonitrile:methanol (85:15) was used at different time intervals. The flow rate was 1.5 ml/min and effluent was monitored at 218 nm. The retention times of montelukast sodium and bambuterol hydrochloride were 21.2 min and 5.8 min respectively. The linearity for both the drugs was in the range of 0.25-0.75 mg/ml with correlation coefficients of 0.9999 and 0.9996 for montelukast sodium and bambuterol hydrochloride, respectively.

Montelukast sodium (MTK), 1-[({(R)-m-[(E)-2-(7-chloro-2-quinolyl) vinyl]-α-[o-(1-hydroxyl-1-methylethyl)phenethyl]benzyl}thio)methyl]cyclopropaneacetate sodium is a leukotriene receptor antagonist, used in the treatment of asthma[1–3]. It is not official in IP and BP. Various analytical methods, such as liquid chromatography with fluorescence detection[4–6], stereoselective HPLC for MTK and its S-enantiomer[7], simultaneous HPLC and derivative spectroscopic method with loratadine[8], stability indicating HPLC method for MTK in tablets and human plasma[9] have been already reported.

Bambuterol hydrochloride (BBL), (RS)-5-(2-tert-butylamino-1-hydroxyethyl)-m-phenylene bis(dimethylcarbamate) hydrochloride is a direct acting sympathomimetic with predominantly -adrenergic activity (β_2_-agonist)[10]. It is an ester prodrug of β_2_ adrenergic agonist terbutaline[11]. Bambuterol hydrochloride is official in BP[12]. Different HPLC methods have been reported for the estimation of BBL in pharmaceutical dosage form[13–15]. The drug has been also estimated by solid-state NMR spectroscopy[16]. The combination dosage forms of MTK and BBL are available in the market for the prophylaxis and treatment of chronic asthma and chronic bronchitis in pediatrics. Present study involves development and validation of RP-HPLC method for the estimation of MTK and BBL in combination dosage form.

Combination tablet formulation containing montelukast sodium equivalent to montelukast 10 mg and bambuterol hydrochloride 10 mg (Montair Plus, Okasa Pharma, Satara, India) was procured from the local pharmacy. HPLC grade acetonitrile, methanol (Rankem, India) and HPLC grade water (Milli-Q) were used in this method. NaH_2_PO_4_ was of analytical grade obtained from Qualigens (India). Mobile phase A was prepared by mixing 850 ml of 0.025M NaH_2_PO_4_ buffer with 150 ml of methanol and mobile phase B was prepared by mixing 850 ml of acetonitrile with 150 ml of methanol. The solution was sonicated for 10 min and filtered using Whatman filter paper (No.41).

A Shimadzu HPLC LC-2010 AHK unit and Agilent 1100 system with variable wavelength programmable UV/Vis detector, an inertsil ODS C-18, 5 μm column of dimensions 250×4.6 mm was used. A Rheodyne injector with a 10 μl loop was used for the injection of sample.

Standard stock solution was prepared by weighing pure MTK and BBL (25 mg each) and dissolving in 30 ml of diluent in 50 ml volumetric flask. The solution was sonicated for 15 min, cooled and volume was made up to the mark with diluent to obtain final concentration of 500 μg/ml each. The solution was filtered. Calibration curves were prepared by taking appropriate aliquots of standard MTK and BBL stock solution in 10 ml volumetric flask and diluted up to the mark with diluent to obtain final concentrations of 250, 300, 400, 500, 600, 700, 750 μg/ml of each. Standard solutions (n=6) were injected through 10 μl loop system, and chromatograms were obtained using 1.5 ml/min flow rate. The time programme was set for gradient elution. Different compositions of mobile phases at different time intervals (mobile phase A:mobile phase B, 85:15 at 0 min, 15:85 after 15 min, 15:85 after 22 min, 85:15 after 28 min and 85:15 after 33 min) were run to obtain the satisfactory resolution. The effluent was monitored at 218 nm. Calibration curve was constructed by plotting average peak area against concentration, and regression equations were computed.

Five intact tablets (0.9380 g) containing MTK and BBL, each of 10 mg, were weighed accurately and transferred to 100 ml volumetric flask, sonicated for 15 min and the volume made up to the mark with diluent (water:acetonitrile:methanol, 1:1:1) to obtain final concentration of 500 μg/ml of each drug. The solution was filtered. Sample solutions were chromatographed (n=6), and concentrations of MTK and BBL in tablet samples were found using regression equations.

The average retention time for MTK and BBL was found to be 21.2 min (% RSD, 0.28) and 5.8 min (% RSD, 0.15), respectively (fig. 1). The linearity of the assay was checked at 50-150% of the assay level concentration of MTK and BBL. The calibration was linear in the range of 0.25-0.75 mg/ml for both the drugs with regression coefficient 0.9999 and 0.9996, intercept −24564.35 and 69825.13 and slope 22166620.23 and 8402793.74 for MTK and BBL, respectively. The low % RSD value of peak area, 0.32 (MTK) and 0.19 (BBL) indicated that the method is precise and accurate (Table 1).

**Fig. 1:**
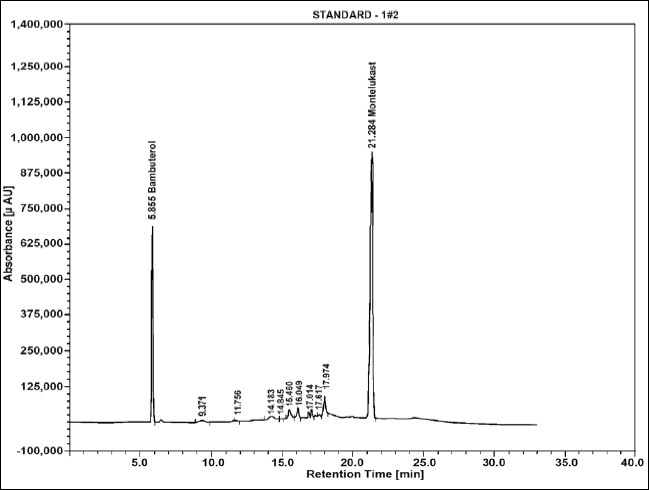
typical chromatogram for montelukast and bambuterol

**TABLE 1:** LINEARITY AND PRECISION OF HPLC METHOD

Parameter	MTK	BBL
Correlation coefficient	0.9999	0.9996
Slope	22166620.23	8402793.74
Intercept	- 24564.35	69825.13
% RSD of area of standard	0.32^a^	0.19^a^
% RSD of retention time (min) of standard	0.21^a^	0.09^a^

a Values of % RSD of six estimations; RSD: Relative standard deviation.

The content of MTK and BBL were determined using regression equation of standards. The % drug content was found to be 101.3±0.66 for MTK and 98.56±0.82 for BBL. Recovery studies were carried out at 50%, 100% and 150% level. The mean recoveries (n=3) were found to be 99.45-99.97% (% RSD 0.56-1.92) for MTK and 99.76-100.3% (% RSD 0.76-1.87) for BBL. The low % RSD values obtained for repeatability (n=6), intra-day (n=3), inter-day variation (n=3) and robustness (n=3) indicated that the method was precise.

An accurate, specific and precise assay using level gradient reverse-phase high-performance liquid chromatographic procedure for the simultaneous determination of MTK and BBL in tablets was developed in the present investigation. Satisfactory separation was obtained with the gradient system. The results obtained by the proposed method were close to the label claim of both the drugs. The low value of % RSD and recovery experiments indicates that the method is accurate.
